# MicroRNAs in *C. elegans* Aging: Molecular Insurance for Robustness?

**DOI:** 10.2174/138920209788185243

**Published:** 2009-05

**Authors:** Carolina Ibáñez-Ventoso, Monica Driscoll

**Affiliations:** Department of Molecular Biology & Biochemistry, Rutgers, The State University of New Jersey, A232 Nelson Biological Laboratories, 604 Allison Road, Piscataway, New Jersey 08854, USA

**Keywords:** miRNA, *Caenorhabditis elegans*, sarcopenia, lipofuscin, healthspan, longevity.

## Abstract

The last decade has witnessed a revolution in our appreciation of the extensive regulatory gene expression networks modulated by small untranslated RNAs. microRNAs (miRNAs), ~22 nt RNAs that bind imperfectly to partially homologous sites on target mRNAs to regulate transcript expression, are now known to influence a broad range of biological processes germane to development, homeostatic regulation and disease. It has been proposed that miRNAs ensure biological robustness, and aging has been described as a progressive loss of system and cellular robustness, but relatively little work to date has addressed roles of miRNAs in longevity and healthspan (the period of youthful vigor and disease resistance that precedes debilitating decline in basic functions). The *C. elegans *model is highly suitable for testing hypotheses regarding miRNA impact on aging biology: the lifespan of the animal is approximately three weeks, there exist a wealth of genetic mutations that alter lifespan through characterized pathways, biomarkers that report strong healthspan have been defined, and many miRNA genes have been identified, expression-profiled, and knocked out. 50/114 *C. elegans *miRNAs change in abundance during adult life, suggesting significant potential to modulate healthspan and lifespan. Indeed, miRNA *lin-4 *has been elegantly shown to influence lifespan and healthspan *via *its *lin-14 *mRNA target and the insulin signaling pathway. 27 of the *C. elegans *age-regulated miRNAs have sequence similarity with both fly and human miRNAs. We review current understanding of a field poised to reveal major insights into potentially conserved miRNA-regulated networks that modulate aging.

## INTRODUCTION

microRNAs (miRNAs) are short non-translated RNAs (~22nt) that regulate post-transcriptional gene expression *via *antisense base pairing to partially complementary sites in the 3’ UTRs (primarily) of messenger RNAs (mRNAs). miRNAs downregulate protein expression by inhibiting mRNA translation and/or mRNA stability, although induction of rapid degradation of nascent peptides has also been proposed as a regulatory mechanism [1], and there is at least one reported case of translational upregulation by components of the miRNA machinery [2]. Individual miRNAs can modulate multiple mRNA targets, and individual mRNAs can be regulated by multiple, distinct miRNAs. The picture that emerges is one of highly complex regulatory networks that control batteries of gene targets. Of the thousands of miRNAs catalogued in the miRNA repository miRBase [3-5], functional characterization of a small fraction supports the participation of miRNAs in a broad range of biological processes, including cell proliferation, morphogenesis, cell differentiation, metabolism, immunity, stress response, signalling, cell death, cancer and age-related disease such as neurodegeneration. More recently, several groups have turned to consider the possible roles of miRNAs in the biology of aging. Here we review initial efforts in this direction in the powerful* C. elegans* model, which holds distinct advantages for experimentally addressing the importance of miRNAs in genetic modulation of longevity and healthspan.

## *Caenorhabditis elegans* AS A MODEL SYSTEM FOR STUDYING ROLES OF MicroRNAS IN AGING AND HEALTHSPAN

The nematode *C. elegans* is a genetically tractable model organism of uniform genetic background that has been extensively used in the study of aging and longevity to identify genes that impact these processes. This small (1mm long) transparent animal develops in ~ 3 days through a reproducible set of fully documented cell divisions to generate a sexually mature adult of 959 somatic cells. *C. elegans* somatic cells do not undergo any regenerative cell divisions and developmental cell divisions are completed during larval development. Thus, the aging biology of this animal best models the situation in mammalian tissues that do not divide to replenish or repair cells. The average *C. elegans* lifespan is about 21 days under standard lab conditions (abundant food, 20°C), although lifespan can be dramatically changed by environmental factors such as low temperature growth or food limitation (dietary restriction). Notably, there is a considerable degree of variation in mean and maximum lifespan within a genetically uniform population reared under the same conditions, indicating that stochastic factors have a significant impact on healthspan and lifespan [6].

In the lab at 20°C, *C. elegans* life can be described as three phases: 1) a period of development to sexual maturity (egg through 4 larval stages, about 3.5 days); 2) the reproductive period (reproduction by self-fertilization occurs for the first 5-6 days of adult life until sperm supplies are exhausted, although mating can result in additional progeny production even fairly late in life [7]); and 3) a post-reproductive phase (~two weeks). It is striking that the decline over adult life bears many features of aging in other animals, many of which live considerably longer. This suggests that similar processes and mechanisms modulate age-associated decline across phyla [8]. For example, *C. elegans* locomotory behaviors of crawling on plates or swimming in liquid decline in vigor with age. This decline is correlated with physical deterioration of the bodywall muscle cells in a manner that closely resembles human sarcopenia [6], a condition of progressive loss of muscle mass and muscle strength that begins with mid-life onset and progresses to be highly debilitating at advanced age [9, 10]. Pumping of the pharyngeal muscle, proposed to be analogous to cardiac muscle, also becomes progressively slower and erratic with age [11, 12]. As in mammalian non-dividing cells, autofluorescent advanced glycation end (AGE) products and lipofuscin accumulate with age [13, 14]. These age pigments are a heterogeneous mix of cross-linked proteins, lipids and nucleic acids altered by attached reactive sugars that have been proposed to impair lysosomal function. In *C. elegans* low age pigment accumulation is correlated with "graceful" aging and extended healthspan and lifespan; conversely, high age pigments are correlated with physiologically aged, decrepit animals [14]. The point to be made here is that in the nematode model, there are several biomarkers that reflect the quality of aging, and these can be measured to identify animals that age better than others.

More than 300 genetic manipulations in *C. elegans* have been reported to affect lifespan [15], a clear demonstration that genes influence longevity. A major pathway impacting* C. elegans* lifespan and healthspan is a conserved insulin/IGF-like signal transduction pathway that was first characterized for its role in formation of dauer larvae under adverse growth conditions. Reduction-of-function mutations in the *daf-2* insulin/IGF-1 receptor and downstream signaling kinases can confer longevity [16-19] by releasing DAF-16/FOXO transcription factor from inhibitory phosphorylation to activate beneficial transcription [20-23]. In addition to genes regulating insulin signaling, genes influencing developmental timing, sensory signaling, mitochondrial function, and dietary restriction can significantly alter *C. elegans *lifespan [24]. Mutations affecting specific processes can be combined in double mutants to produce additive effects on longevity. Record mean and maximum longevity (~ 10X) has been reported for null mutants of *age-1* PI3 kinase [25], but standard lifespan extension of single gene mutations is more commonly on the order of 20-50%. In general, research has focused on mutations or RNAi knockdowns that confer lifespan extension rather than short life, because of the challenges of unambiguously distinguishing sickness from early aging. Recent studies that better define healthspan and aging indicators such as locomotion decline [6, 11, 26], accumulation of lipofuscin and age pigments [13, 14], pharyngeal pumping [11, 12], and even gene expression profiling [27], now make it easier to distinguish nematode progeria (accelerated aging). Given the short lifespan, multiple indicators of healthy aging, and a wealth of available genetic reagents, the *C. elegans* model appears optimally poised to be exploited to address roles of miRNAs on lifespan and healthspan.

### A Snapshot View of the microRNAs from a Model Organism

According to the current annotation of miRBase (release 12.0), there are 154 identified* C. elegans *miRNA genes that encode sequences with characteristic miRNA precursor features (one strand with the potential to encode imperfectly base-paired hairpin structures that can be processed into ~22 nt mature miRNA [28]). Given that the *C. elegans* genome sequence has been exhaustively analyzed for potential *mir *genes, and that extensive deep sequencing has been reported for this animal [29, 30], it is likely that the current list represents nearly all of the miRNAs expressed under standard growth conditions. Although more *C. elegans mir* genes may be discovered by biochemical approaches (as in ref. [31]), or in deep sequencing studies under specific growth regimens, the current list is likely to constitute a fairly accurate overview of the miRNA content of an entire organism under the conditions in which most experimental investigations are conducted.

What does a genome-wide overview of miRNAs reveal with regard to numbers of distinct miRNA families and their potential for functional redundancy? We evaluated all sequence relationships of mature *C. elegans* miRNAs in miRBase release 10.1 [32]. We classified miRNAs as potentially functionally redundant based on 5’ end “seed” sequence similarity and/or ≥70% similarity extended over the full (~22 nt) mature sequence because miRNAs related by either of these criteria have been experimentally demonstrated to have functional redundancy [33-36]. In general, ~60% of *C. elegans* miRNAs share significant sequence similarities with distinct miRNAs encoded elsewhere within the nematode genome. These sequence relationships suggest considerable potential for functional redundancy within families, although evaluation of physiological redundancy requires addressing the question of whether related miRNAs are co-expressed in the same cell types at the same time, a project well underway [37]. One important consequence of potential functional redundancy among miRNAs is that genetic knockouts of individual *mir *genes may not suffice to reveal the biological function of a given family. Indeed, elegantly studied examples of miRNA functional redundancy have been published for the role of the *let-7* family members in regulating timing of developmental processes [33, 36]. The potential for functional redundancy may partially explain the general observation from evaluation of 92 genetic knockouts of C. elegans mir genes that most individual mir deletions do not confer readily apparent phenotypes under standard growth conditions [38]. Thus, studies that evaluate aging phenotypes in mir mutants will need to take potential functional redundancy into consideration.

### Many *C. elegans* microRNAs are Conserved Across Species

Another important question germane to use of genetic models to inform on miRNA functions in the biology of aging regards how well miRNA sequences and functions might be conserved in evolution. miRNAs pose interesting problems in evolutionary biology because sequences need to be maintained in both the miRNA as well as in the critical target transcripts; miRNAs standardly modulate expression of multiple targets, adding to potential constraints [39]. This being said, sequence conservation of miRNAs across species is considerable. In miRBase 10.1, ~62% of *C. elegans *miRNAs are related to *Drosophila* miRNAs and ~55% of *C. elegans* miRNAs are related to human miRNAs—more than half of *C. elegans* miRNAs have common sequences in both flies and humans [32]. This extensive conservation suggests that miRNAs could exert common functional roles across phyla. One striking example of such a case is *let-7* family regulation of RAS oncogene expression. *C. elegans let-7* miRNA regulates the timing of fate differentiation of multipotent seam cells during *C. elegans* development [40], and sequence-related miRNAs *let-7* and miR-84 have been shown to negatively regulate *C. elegans *RAS gene *let-60*, which contributes to developmental signaling [36]. In humans, *let-7* family *mir* genes are poorly expressed in a range of malignancies including lung cancer [41, 42]. *In vitro*, human *let-7* has been shown to down-regulate expression of RAS protein and over-expression of *let-7* inhibits the growth of human lung cancer cells [36, 42]. Recent over-expression studies in a mouse K-RAS lung cancer model demonstrated tumor suppressor function for *let-7* *in vivo* [43], a result with clear therapeutic implications. Thus, dissection of basic biology of a miRNA family in an experimentally accessible model can inform on conserved functions. Whether this will be true in the biology of aging remains to be tested.

Overall, however, the *C. elegans* model, with the plethora of molecular and genetic manipulations that can be executed, the comprehensive identification of conserved miRNAs that has been accomplished [32], the wealth of available miRNA deletion mutants [38], records of spatiotemporal expression patterns for many canonical miRNAs [37, 44-47], and the computational predictions of miRNA targets [48] within a well annotated genome [78] (which can be complemented with target predictions in the nematode using other algorithms [5, 30, 49-53]), is a powerful experimental vehicle for deciphering functions that might apply to the understanding of miRNA functions in aging organisms.

## *C. elegans* MicroRNAs CHANGE EXTENSIVELY IN ABUNDANCE DURING ADULT LIFE

How miRNAs might regulate healthspan and lifespan is a question that is just coming into focus and is likely to remain at the forefront of aging research for some time. As a first step toward understanding how miRNAs might impact aging, we documented how miRNAs change in abundance during adult *C. elegans* life [48], the first whole-organism miRNA profiling accomplished for aging animals. We isolated small RNAs from aging *C. elegans* adults and detected expression levels of the 114 *C. elegans* miRNAs registered in miRBase 5.0 *via *microarray hybridization [54]. The *C. elegans* strain used for this analysis was rendered sterile by a temperature-sensitive fertility mutation so that age-synchronized cultures could be easily maintained. Fig. (**1A**) depicts sampling time points relative to the lifespan curve. For each age, we normalized miRNA levels to levels in day4 first-day adult animals and plotted values over time. Fig. (**1B**) gives an example of how conserved miRNAs *let-7*, *lin-4* and miR-1 change over adult *C. elegans* life.

Of the 114 miRNAs screened, we found that 50 change in expression during adulthood with a 90% confidence statistic--we refer to these 50 as age-regulated miRNAs. 34 of these age-regulated miRNAs fell within a 95% confidence level. Nearly two thirds (31) varied more than twofold between maximum and minimum expression levels over adult life. In terms of expression patterns, we did not note a clear correlation in the expression of age-regulated miR family members or in that of age-regulated miRNAs clustered in the genome—in some cases family members or clustered *mir* genes appear to have similar expression patterns, but in many they do not.

It is probably worth emphasizing that this *C. elegans* study provides an indication of how whole-body levels of individual miRNAs vary on average, but that miRNAs that change in only a few cells, or miRNAs that change reciprocally in different tissues could be missed in this analysis. Thus, there is likely even more extensive modulation of miRNA levels during aging than appears from this first general survey. Nevertheless, one certain conclusion emerges: there are substantial changes in levels of many miRNAs during adult *C. elegans* life. As such, these age-regulated miRNAs are candidate modulators of aging and longevity.

## CONSERVED AGE-REGULATED MicroRNAs

Interestingly, of the 50 miRNAs that significantly change in expression during *C. elegans* adulthood, 27 share sequence conservation with human miRNAs (Table **1**; refer to [32] for details of sequence relationships). Several of these (for example, *let-7*, miR-1, miR-34) have been investigated for roles in biology apart from aging. Which age-regulated miRNAs influence aging in nematodes, flies and/or humans remains mostly to be experimentally determined. Given that conserved mechanisms such as insulin signaling, dietary restriction and oxidative stress responses impact both lifespan and healthspan across phyla, it is plausible that functional studies of conserved miRNAs in facile invertebrate models could identify a novel set of small molecule modulators of universal components of aging.

## DO PATTERNS OF MicroRNA CHANGES REVEAL COMPONENTS OF AGING BIOLOGY?

We found that miRNA expression levels varied in a range of pattern types during *C. elegans* adulthood. Interestingly, a considerable number of age-regulated miRNAs diminish in expression over adult life. This trend is most striking when considering expression changes during the reproductive phase of early adulthood (days 6-8) or from early to mid adult life (days 6-11). Expression of about two thirds of the age-related miRNAs (32/50 p≤0.1; 23/34 p≤0.05) diminishes during the reproductive stage (d6-8) or during the reproductive to mid-life stage (d6-11). This downregulation is not likely to be solely attributed to germ cell production since germ cell proliferation would increase miRNA abundance, and because many of the miRNAs that change with age do not appear to be germline expressed in published studies. Expression of most of these age-regulated miRNAs is similar in wild-type and young *glp-4(bn2)* mutant adults that lack most germline cells [44], indicating that expression changes most likely reflect modulation in the adult soma rather than the lack of developing embryos in the *spe-9* mutant (our study used mutations to block fertilization so embryonic development cannot contribute). That there appears to be a general down-regulation of miRNAs suggests that a relaxation in precise transcriptional/translational controls might be a general feature of advancing adult life in *C. elegans*. It is conceivable that a modest dysregulation of protein synthesis could initiate declines in multiple systems during adulthood. This hypothesis remains to be tested, beginning with whole-genome analysis of proteomic consequences of miRNA decline.

**Fig.(1). Representative miRNA expression profiles over C. elegans adult life.**
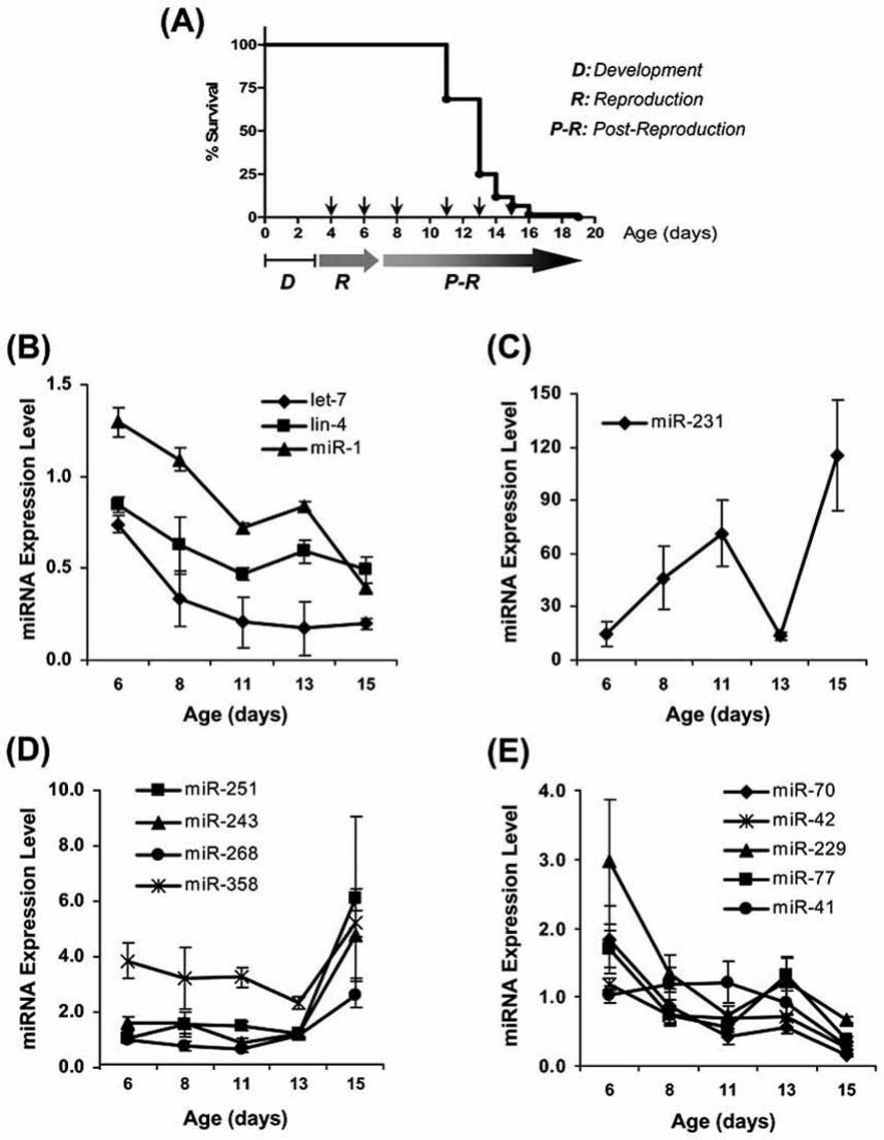
**A: Lifespan of *C. elegans spe-9(hc88)* in one representative sample collection for small RNA isolation (n=60, 25.5 °C).** Time 0 corresponds to harvest of eggs and reproductive period extends over days ~3-7 (judged from laying of unfertilized oocytes in this fertility mutant). Small RNA samples were prepared from days marked by downward arrows (days 4, 6, 8, 11, 13 and 15). **B: Expression of conserved microRNAs *let-7, lin-4* and miR-1 over adult life**. Expression levels are normalized to the levels measured at day 4, the first day of adult life. A downward trend in expression is typical of most age-regulated miRNAs. **C: Expression of microRNA miR-231 over adult life**. Expression levels are normalized to the levels measured at day 4. miR-231 is one of a few age-regulated miRNAs that generally increases with age and is also the most abundantly expressed. It might be noteworthy that the 25% survival point of 13 days often appears to be a transition point in expression profiles for individual miRNAs. **D: microRNAs showing highest expression late in life (day 15) from the 95% confidence group of 34 age-regulated microRNAs**. Expression levels are normalized to the levels measured at day 4. miR-231 (Fig. 1C) also shows greatest expression on day 15. **E: microRNAs showing lowest expression late in life (day 15) from the 95% confidence group of 34 age-regulated microRNAs.** Expression levels are normalized to the levels measured at day 4.

One can speculate that specific patterns of expression might identify miRNAs that exert specific influences on the biology of aging. For example, miRNAs that decline early in adult life might be associated with, or possibly even causal in, “mid-life” crises in metabolism such as the transition to increased rates of age pigment/lipofuscin accumulation or the onset of sarcopenia [6, 14]. The miRNAs that exhibit highest or lowest levels at the end of life (see Fig. **1D-E** and Suppl. Fig. S2 in [48] for a list of 25 that exhibit greatest changes at the last sampling time) could reflect two alternative situations. On the one hand, on the last sampling day (15 days), 93% of animals have already died and the animals that are still viable are all in the most decrepit stage of life. Thus, the miRNA profile at this point might best reflect the end-stage, extremely aged animal. Alternatively, one could note that the surviving 7% of the population have lived longer than most of the culture and thus they might express a miRNA profile more typical of the most robust animals in a given culture—the *C. elegans* equivalent of centenarians. If any of the late-changing miRNAs identified are causative in end-stage aging biology, these two models might be experimentally addressed by testing consequences of deletion or over-expression of miRNAs that change the most at the day 15 timepoint. Finally, one might wonder about the miRNAs with greatest changes in relative abundance over adult life. One miRNA, conserved miR-231 (Fig. **1C**), stands out as the most abundant age-regulated *C. elegans* miRNA (highest levels at day 15) with the largest net increase (~11-fold) and greatest variation over adulthood (big increase to day 11, sharp decrease at day 13 and strong increase at day 15). miR-231, and other miRNAs that change with age, might impact expression of gene batteries that modulate lifespan or the quality of aging, a general hypothesis that can be tested with the powerful research tools that can be applied in this model. We list age-regulated *C. elegans* miRNAs exhibiting largest changes in expression in (i) early adulthood, (ii) late adulthood or (iii) over adult life in Table **2**.

## MicroRNA *lin-4* MODULATES THE QUALITY OF AGING AND THE LIFESPAN OF *C. elegans*

A key question is to what extent miRNAs influence the biology of healthy aging and lifespan in a given organism and across species. It has been proposed that miRNAs function in biology to insure developmental robustness and maintain homeostasis [55]. Since aging has been described as a progressive loss of robustness and stress resistance, a logical corollary is that miRNA changes during adult life might limit robustness and contribute to aging. Analysis of *lin-4* impact on aging [56] is the first example of a miRNA that plays a defined role in normal development and also modulates both healthspan and lifespan.

**Fig. (2). Summary of microRNA lin-4 and target lin-14 relationships in developmental cell fate specification and in longevity.**
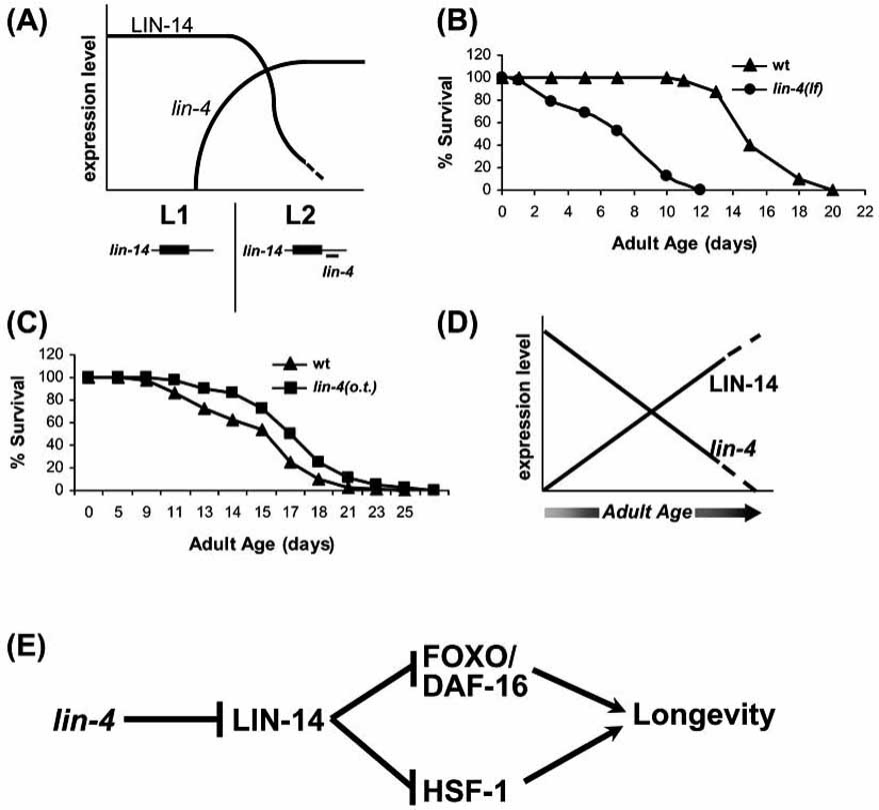
**A: Representative schematic of *lin-4_lin-14* interaction with regard to their expression changes during early larval development.** The miRNA *lin-4* increases in expression level at the end of the first larval stage (L1), when it down-regulates expression of *lin-14* transcript and consequently LIN-14 protein to allow second larval stage (L2) cell fates to occur. For depicted *lin-14* transcript, box indicates coding region and flanking lines indicate UTRs. Only one of the seven *lin-4* complementary sites in the 3’ UTR of the *lin-14* transcript is shown. **B-C: Survival trends of *lin-4* loss-of-function (lf) mutant (B) and *lin-4* over-expressing transgenic (o.t.) (C) compared to those of wild type (wt)**. Please refer to [56] for actual graphs and data. **D: Proposed *lin-4*_LIN-14 expression relationship during adulthood.** *lin-4* miRNA levels fall during midlife, which could increase expression of LIN-14 protein. **E: Diagram summary of *lin-4*_LIN-14 effect on longevity through FOXO/DAF-16 and HSF-1.** Low levels of *lin-14* expression allow enhanced expression of lifespan-promoting FOXO/DAF-16 and HSF-1 transcription factors [56].

During development, *C. elegans* *lin-4* binds to 3’ UTR sequences of the *lin-14* mRNA to downregulate expression of the transcription factor LIN-14, enabling specification of hypodermal cell fate transition from the first to the second larval stage [57] (Fig. **2A**). With regard to adult phenotypes, *lin-4* loss-of-function (lf) shortens lifespan, whereas over-expression of *lin-4* lengthens lifespan (Fig. **2B-C**). Long life is associated with lowered levels of age pigment accumulation and increased heat stress resistance, suggesting that not just lifespan, but also healthspan is modulated by the *lin-4* miRNA. In support of a normal role for *lin-4* in healthspan promotion, we have also observed accelerated locomotory decline in middle-age *lin-4(lf)* mutants (C.I-V, MV and MD unpublished observations). *lin-4* requires the presence of target *lin-14* to exert its effects in adult life, with *lin-14(gf)* (a mutant that lacks the *lin-4* binding sites in the 3’UTR) mimicking the deleterious effects of *lin-4(lf)* [56]. Together, these observations suggest that normal *lin-4* function in adult life limits pro-aging effects of *lin-14*. Since *lin-4* is one of the miRNAs that decline in abundance with age, we could speculate that as *lin-4* levels diminish during adult life, elevated expression of *lin-14* occurs in aging animals with deleterious consequences for the quality of aging (Fig. **2D**). This may be a good example of antagonistic pleiotropy in which a gene that confers developmental benefit is actually deleterious later in life [58].

How does *lin-14* promote age-associated decline? *lin-14(lf)* does not extend lifespan without the critical downstream transcription factors of insulin signaling FOXO/DAF-16 or HSF-1, suggesting that *lin-14* modulates the level of insulin signaling, a well characterized mechanism for modulation of lifespan (Fig. **2E**).

## ROLES OF OTHER *C. elegans* MicroRNAs IN AGING

Many *C. elegans* *mir* genes have been knocked out, including 87 deleted in one massive screen [38]. Interestingly, the majority of these *mir *deletion mutants did not exhibit lethality or major phenotypic changes in developmental timing, mobility, gross morphology and reproduction. One possible explanation is that there could be a considerable amount of functional redundancy among sequence-related or other miRNAs. As noted above, there is strong experimental evidence for functional redundancy for the *let-7* family [33, 36], and many *C. elegans* miRNAs share potential for functional redundancy [32]. It is also possible that miRNAs primarily modulate protein expression levels such that the consequences of their individual disruption are not dramatic. Apart from the elegant *lin-4* investigations on lifespan [56], there are no published reports yet on roles of other *C. elegans *miRNAs on lifespan and healthspan. Although much carefully controlled work remains to be done, a first pass look at age-associated phenotypes in several *mir* mutants suggests that multiple miRNAs may prove to promote healthspan (CIV, MV, SG, MS, DZ and MD, unpublished observations), consistent with the hypothesis that this gene class may contribute to robustness required for maintenance of healthspan. If this proves to be the case, the *C. elegans* model will be an important proving ground for genetic miRNA “therapies” that might promote healthy aging in transgenic studies.

## WHAT ARE THE TRANSCRIPTS IMPACTED BY AGE-REGULATED MicroRNAs?—THE CHALLENGE OF TARGET PREDICTIONS

Central to the understanding of miRNA impact on aging will be the identification of physiologically relevant targets. The expectation is that a highly complex web of miRNA interactions must exist, given that individual transcripts can be targeted by multiple miRNAs and that individual miRNAs can interact with multiple transcripts. In the absence of quantitative, cell-specific biochemical identification of miRNA-targeted transcripts, computational target predictions have been generated to suggest the arrays of mRNA transcripts that might be subjected to regulation by specific miRNAs (e.g. [48, 53, 59, 60]). It should be underscored that different target prediction algorithms do not necessarily predict the same target lists, although programs are continuously being improved based on accumulating experimental data [61, 62]. Thus, the predictions are taken to suggest potential targets, but few targets have been experimentally verified and modeling based on these predictions should be extremely cautious until verifications are established.

We used an algorithm described in [63] to search all predicted *C. elegans* transcripts for 3’ UTR target sites of the 50 age-regulated miRNAs (Suppl. Table 2 in [48]). We also generated specific target group lists to ask whether, in principle, the age-regulated miRNAs might preferentially target genes previously implicated in longevity, or to identify potential mRNA targets of interest for aging biology. For example, we screened a list of more than 200 genes known to influence *C. elegans* lifespan (referred to as gerontogenes; 204 listed in Suppl. Table S3 of [48]) to identify 42/204 gerontogenes that have predicted 3’UTR binding sites for age-regulated miRNAs. Conversely, we find 31/50 age-regulated miRNAs are predicted as candidate modulators of these gerontogenes. When we considered the group of genes involved in insulin signaling, we found that age-regulated miRNAs might target 14/39 insulin transcripts and that 11 of the 50 age-regulated miRNAs potentially target insulin genes. We list the gerontogenes and insulin genes that have multiple predicted sites for age-regulated miRNAs in Table 3 (see [48] for total lists; miRNA *lin-4* influences aging through regulation of 7 sites in the 3’ UTR of the gene *lin-14* [56, 57, 64]).

Computational strategies such as these can highlight specific miRNAs for experimental investigations of tissue-specific age-related declines. For example, with an interest in identifying miRNAs that might impact muscle aging, we searched a list of genes expressed preferentially in muscle [65] for 3’ UTR sites that might bind age-regulated miRNAs. We found that of 1364 muscle-enriched genes, 232 have potential 3’ UTR binding sites for age-regulated miRNAs. Age-regulated miR-1 (17), miR-268 (38), and miR-85 (31) have the most predicted targets in the muscle-enriched gene list and thus could be prioritized for testing for roles in sarcopenia.

## MicroRNA CHANGES IN MAMMALIAN MODELS OF AGING

Gigantic strides have been accomplished in the understanding of miRNA biogenesis and in deciphering mechanisms of miRNA regulation of cellular processes during development, health and pathology. Mammalian miRNA profiles in the areas of diseases of aging such as cancer [66] and neurodegeneration [67] have been reported. The investigation of miRNAs in aging and age-related diseases is just beginning to come into focus--analyses of miRNAs in aging muscle [68], liver [69], and brain [70] have been recently published to highlight miRNA expression changes during adult life; by contrast, miRNAs in aging lung do not appear to change [71]. Already, studies in aging subjects have provided unexpected insights into miRNA regulation in older animals. For example, analysis of miRNA profiles in exercised skeletal muscle [68] suggests that aging might be correlated with differences in precursor, rather than mature, miRNA levels, a fact that might have physiological consequences.

At present, it is probably premature to compare mammalian tissue-specific studies to whole animal surveys in *C. elegans*. However, intriguing potential connections have begun to appear. For example, it is tantalizing that in mammalian cells, miR-34 elevation is associated with p53-induced cell senescence [72, 73] and *C. elegans* miR-34 is one of the few miRNAs that increase in abundance with age [48]. Hypotheses regarding potential miR-34 roles in promoting senescence in *C. elegans* could easily be tested given reagents available in this facile model. In the future, key advances in cataloging age-regulated miRNAs in mammalian tissues should be compared with studies of roles of *C. elegans* miRNAs in analogous tissues to begin to address whether conserved miRNA-regulated processes relevant to aging biology might exist.

## PERSPECTIVES

In this review we have emphasized how basic characterization of miRNAs in *C. elegans* converge with extensive lifespan and healthspan studies conducted in this model to date to set up a powerful system for the experimental evaluation of roles of miRNAs in the biology of aging of a single organism. We know that there are significant changes in miRNA abundance during adult life that occur in various “patterns” (with nearly 50% of 114 tested miRNAs modulated) and the elegant *lin-4* study from Bohem and Slack [56] serves as a proof-of-principle example that a single conserved miRNA can modulate lifespan and healthspan. Major questions, however, remain to be addressed. We have suggested that miRNAs contribute to the robustness of the animal and that the general decline in miRNA levels that is apparent when expression in all tissues is averaged over adult life might contribute to age-associated deterioration and dysfunction. How broadly miRNAs are implicated in lifespan and healthspan modulation can now be addressed in the nematode model by systematic evaluation of lifespan and healthspan indicators in *mir* mutants and in lines that over-express individual *mir* genes. It will be particularly important to determine whether there a few key “node” miRNAs that regulate large networks of genes that impact aging, such as oxidative stress response networks, metabolic pathways, and insulin signaling pathways. Defining such node miRNA regulators of aging biology will hold particular significance if they exert conserved roles in mammalian healthspan.

It is also possible that specific miRNA levels in adult life have potential to be used as diagnostics for scoring “physiological” (rather than chronological) age as is the case for age pigment levels in *C. elegans* [14]. Comparisons of miRNA profiles in animals that age well *vs* those that age poorly might indicate expression patterns correlated with strong healthspan. Given the suggestion that miRNA changes in midlife might underlie some of the early pre-clinical changes that ultimately manifest as age-associated decline [74], it should be possible to address this hypothesis by genetic manipulations of miRNAs that change midlife in the nematode model.

Finally, the *C. elegans* model might also be used to address how well genetic interventions using miRNAs can be used to extend healthy life phases. Genetic engineering or direct miRNA administration that modifies miRNA expression time or level can be conducted to address basic principles regarding small molecular therapeutics that promote healthy aging. Our initial impression is that whole life over-expression of individual *mir *genes may not be easily tolerated by the animal (disruption of balance in developmental roles might be deleterious)—however, late life expression/administration, more relevant to potential clinical application, remains to be evaluated. Success will support conjecture that miRNA modulation might have therapeutic value [75, 76] in mid and late life to improve human healthspan.

## Figures and Tables

**Table 1:** Conserved Age-Regulated MicroRNAs

*let-7*^ 5’ 70^	miR-54 ^5’ Δ^	miR-81 ^5’ Δ^
*lin-4*^5’ 70^	miR-56 ^5’ Δ^	miR-82 ^5’ Δ^
miR-1 ^5’ 70 Δ^	miR-57 ^5’ 70 Δ^	miR-228 ^5’ 70 Δ^
miR-2 ^5’ Δ^	miR-58 ^5’ Δ^	miR-229 ^5’ Δ^
miR-34 ^5’ 70 Δ^	miR-63 ^5’ Δ^	miR-231 ^5’ Δ^
miR-43 ^5’ Δ^	miR-64 ^5’ Δ^	miR-241 ^5’ Δ^
miR-45 ^5’ Δ^	miR-65 ^5’ Δ^	miR-251 ^5’ Δ^
miR-50 ^5’ 70 Δ^	miR-73 ^5’ Δ^	miR-268 ^5’ Δ^
miR-51 ^5’ 70 Δ^	miR-74 ^5’ Δ^	miR-273 ^5’ Δ^

27 *C. elegans* age-regulated miRNAs have conserved sequences in humans, and all of these are also conserved to some extent in *Drosophila melanogaster*. Detailed sequence relationships are described in [32]. Δ indicates miRNAs for which deletion alleles are available; mutant strains carrying point mutations are also available for miRNAs *let-7* and *lin-4* [77]. Superscript numbers inform on the degree of miRNA sequence conservation. **5’:** sequence homology mostly restricted to the 5’ end, **70:** ≥70% sequence identity over miRNA length.

**Table 2:** MicroRNAs that Dramatically Change in Expression Levels Early (i), Late (ii) or Over (iii) *C. elegans* Adult Life (95% Confidence Statistic)

Adult Stage	Expression Levels	
	(A) Increase	(B) Decrease
(i) Early	**miR-34**	**let-7**, miR-36, miR-42, miR-59, miR-77
(ii) Late	**miR-231**, miR-243, **miR-251, miR-268**, miR-358	miR-41, miR-42, miR-70, miR-77, **miR-229**
(iii) Over Adulthood	**miR-34, miR-231,** miR-243, **miR-251, miR-268**	miR-42, miR-70, miR-77, **miR-229, miR-273**

miRNAs with largest increases or decreases in each indicated stage of adult life are included in columns (**A**) and (**B**), respectively. miRNAs within a group are listed in numerical order. Bold miRNAs with homologous sequences among human miRNAs (see Table **1** and [32] for specific details on the sequence conservation).
